# Synergistic Enhancement of Chemiresistive NO_2_ Gas Sensors Using Nitrogen-Doped Reduced Graphene Oxide (N-rGO) Decorated with Nickel Oxide (NiO) Nanoparticles: Achieving sub-ppb Detection Limit

**DOI:** 10.3390/s25051631

**Published:** 2025-03-06

**Authors:** Chiheb Walleni, Mounir Ben Ali, Mohamed Faouzi Ncib, Eduard Llobet

**Affiliations:** 1 MINOS, School of Engineering, Universitat Rovira i Virgili, Avda. Països Catalans 26, 43007 Tarragona, Spain; chiheb.walleni@estudiants.urv.cat; 2 IU-RESCAT, Research Institute in Sustainability, Climatic Change and Energy Transition, Universitat Rovira i Virgili, Joanot Martorell 15, 43480 Vila-seca, Spain; 3 TecnATox—Centre for Environmental, Food and Toxicological Technology, Universitat Rovira i Virgili, Avda. Països Catalans 26, 43007 Tarragona, Spain; 4 Higher School of Sciences and Technologies of Hammam Sousse, University of Sousse, Hammam Sousse 4011, Tunisia; mohamed.faouzi.ncib@gmail.com; 5 NANOMISENE Laboratory, LR16CRMN01, Center of Research on Microelectronics and Nanotechnology (CRMN), Technopole of Sousse, B.P334, Sahloul 4054, Tunisia; mounirbenali@crmn.mesrs.tn; 6 Higher Institute of Applied Science and Technology of Sousse, University of Sousse, Sousse 4003, Tunisia

**Keywords:** N-rGO, NiO, NO_2_, gas sensing

## Abstract

Detecting low nitrogen dioxide concentrations (NO_2_) is crucial for environmental monitoring. In this paper, we report the synergistic effect of decorating nitrogen-doped reduced graphene oxide (N-rGO) with nickel oxide (NiO) nanoparticles for developing highly selective and sensitive chemiresistive NO_2_ gas sensors. The N-rGO/NiO sensor was synthesized straightforwardly, ensuring uniform decoration of NiO nanoparticles on the N-rGO surface. Comprehensive characterization using SEM, TEM, XRD, and Raman spectroscopy confirmed the successful integration of NiO nanoparticles with N-rGO and revealed key structural and morphological features contributing to its enhanced sensing performance. As a result, the NiO/N-rGO nanohybrids demonstrate a significantly enhanced response five orders of magnitude higher than that of N-rGO toward low NO_2_ concentrations (<1 ppm) at 100 °C. Moreover, the present device has an outstanding performance, high sensitivity, and low limit of detection (<1 ppb). The findings pave the way for integrating these sensors into advanced applications, including environmental monitoring and IoT-enabled air quality management systems.

## 1. Introduction

In addition to promoting green energy, safeguarding the environment and human health from harmful gases such as NO_x_, NH_3_, CO_x_, H_2_S, and VOCs is a critical priority in advancing a sustainable lifestyle. Among air pollutants, NO_2_ has garnered significant attention due to its substantial impact on air quality and its association with the formation of ground-level ozone, acid rain, and climate change through disruptions in atmospheric chemical balance. Furthermore, NO_2_ poses serious health risks, including respiratory disorders, chronic bronchitis, asthma, and various cardiovascular and pulmonary diseases, depending on exposure duration [1,2].

To address these challenges, the development of highly sensitive NO_2_ gas sensors has become indispensable. Researchers and industries alike share the responsibility of advancing this technology, which has found increasing relevance in cutting-edge applications such as the Internet of Things (IoT) and Micro-Electro-Mechanical Systems (MEMS) [3]. Effective NO_2_ sensors require several critical attributes, including high selectivity and stability, low power consumption, optimal device architecture, and, most importantly, a carefully chosen sensing material.

Chemiresistive gas sensors have emerged as a preferred choice due to their cost-effectiveness, miniaturization potential, and compatibility with NO_2_ detection [4,5]. Among sensitive materials, graphene-based substances have gained prominence, thanks to their remarkable properties, including a large surface area, high carrier mobility, and excellent electrical and thermal conductivity [6,7,8]. Nitrogen-doped reduced graphene oxide (N-rGO) stands out among graphene derivatives for applications such as energy storage, catalysis, and gas sensing [9,10,11,12]. Its superior performance in gas sensing compared to pristine graphene stems from features like enhanced bandgap engineering, increased active sites for catalytic activity, a high surface area, a defect-rich structure, and low operating temperature. However, a few studies have reported its application in chemiresistive NO_2_ sensors [13,14,15].

In parallel, transition metal oxides (TMOs), known for their semiconducting properties, have demonstrated considerable potential as sensitive materials for chemiresistive gas sensors. TMOs offer advantages such as ease of production, high sensitivity, excellent stability, and rapid response/recovery times [16,17,18]. Despite these benefits, challenges such as high operating temperatures and limited selectivity persist [19,20]. TMOs can be categorized into n-type oxides (e.g., SnO_2_, ZnO, In_2_O_3_, WO_3_, and TiO_2_) and p-type oxides (e.g., NiO and CuO). In gas sensing, n-type oxides typically exhibit resistance changes upon exposure to reducing or oxidizing gases, while p-type oxides demonstrate the opposite behavior [17].

Nickel oxide (NiO), a p-type metal oxide, boasts unique physical properties, including a wide bandgap (3.6–4 eV), excellent thermal conductivity, and stability. These characteristics make it a versatile material for energy storage, optoelectronics, and gas sensing applications [21,22,23]. However, its role as a sensitive material for chemiresistive gas sensors remains relatively rare, even in combination with other materials like graphene derivatives. Studies have suggested synergistic effects when combining NiO with graphene-based materials for detecting gases such as H_2_, H_2_S, NH_3_, CO, and VOCs [24,25,26,27,28]. Nonetheless, its potential for NO_2_ detection under optimized conditions has not been widely discussed.

This study aims to bridge this gap by leveraging the complementary properties of N-rGO and NiO to develop enhanced chemiresistive NO_2_ gas sensors. The sensor materials are synthesized through straightforward methods and evaluated for their performance at sub-ppm NO_2_ concentrations under varying conditions. Their structural and functional attributes are characterized using techniques such as SEM, TEM, XRD, and Raman spectroscopy. Finally, the gas sensing results and mechanisms are thoroughly discussed, highlighting their significance for future applications.

## 2. Materials and Methods

### 2.1. Synthesis of Materials

**Figure 1:**
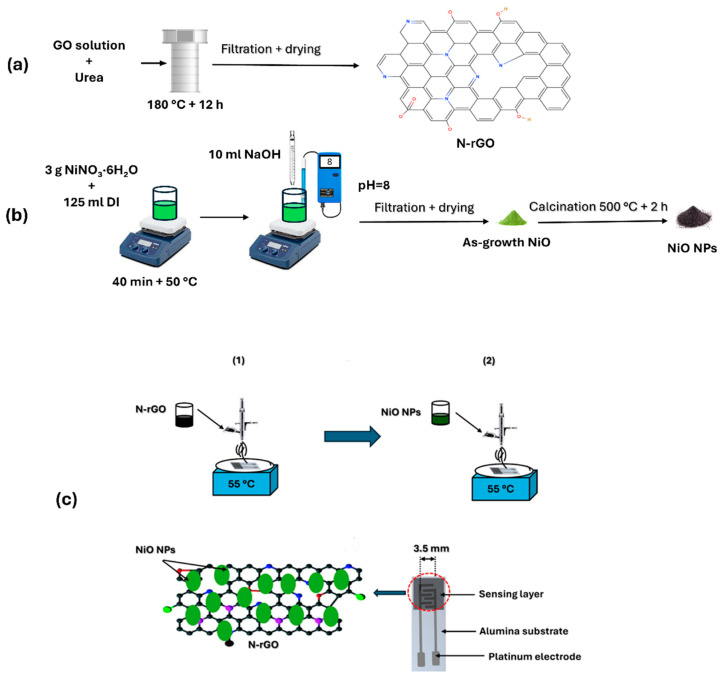
(**a**) Preparation of N-rGO and (**b**) NiO nanoparticles. (1) and (2) illustrate the airbrushing process used to coat the electrode area of the transducer substrate. (**c**) fabrication of the NiO/N-rGO sensor device.

The synthesis of nitrogen-doped reduced graphene oxide (N-rGO) began with the preparation of graphene oxide (GO) as a precursor, which was synthesized using a modified Hummer’s method [29]. In brief, 50 mg of GO powder was dispersed in 50 mL of distilled water and subjected to ultrasonication for 2 h to ensure complete dispersion. Subsequently, an appropriate quantity of urea, serving as the nitrogen dopant source, was added to the GO solution and stirred for 30 min. The resulting mixture was transferred to a Teflon-lined autoclave and subjected to hydrothermal treatment at 180 °C for 12 h. This process not only reduced the GO to reduced graphene oxide (rGO) but also incorporated nitrogen dopants into the graphene structure, yielding N-rGO (Figure 1a).

The synthesis of nickel oxide (NiO) nanoparticles was carried out using the co-precipitation method (Figure 1b). Initially, 3 g of hydrated nickel nitrate (NiNO_3_·6H_2_O) was dissolved in 125 mL of distilled water and stirred at 50 °C for 40 min. Subsequently, 10 mL of 0.1 M NaOH solution was added dropwise to the mixture until the pH reached 8. The resulting precipitate was thoroughly washed and dried at 80 °C. Finally, the dried product was calcined at 400 °C for 3 h to obtain nickel oxide nanoparticles (NiO NPs).

### 2.2. Preparation of Sensors

In this stage, the airbrushing technique was employed to decorate nitrogen-doped reduced graphene oxide (N-rGO) with nickel oxide nanoparticles (NiO NPs). The sensor fabrication process is illustrated in Figure 1c. To begin, 5 mg of N-rGO powder was dispersed in 10 mL of ethanol and subjected to ultrasonication for 1 h to create a stable suspension. Separately, 5 mg of NiO NPs was dispersed in 10 mL of ethanol. The deposition process was carried out using airbrushing at a temperature of approximately 55 °C, with nitrogen (N_2_) serving as the carrier gas. Initially, the N-rGO suspension was airbrushed onto platinum screen-printed electrodes (alumina substrates from CeramTech GmbH, Plochingen, Germany) as a chemiresistive gas sensor. Subsequently, NiO NPs were deposited onto the N-rGO layer to complete the decoration process. The film resistance was monitored continuously using a multimeter, ensuring improved reproducibility across devices. The final thickness of the deposited sensing layers was measured to be 50 ± 5 μm.

### 2.3. Gas Sensing Measurements

In the final stage of the experiment, the as-fabricated NiO/N-rGO sensors were positioned within an airtight Teflon test chamber with a volume of 35 cm^3^, designed with two openings to accommodate UV lamps. The chamber was equipped with an inlet for gas delivery and an outlet connected to an exhaust system. The entire setup was integrated into a fully automated gas flow measurement system capable of supplying diluted gas mixtures via mass flow controllers (Bronkhorst High-Tech B.V., Ruurlo, The Netherlands). For gas sensing experiments, calibrated gas cylinders balanced in dry synthetic air (Air Premier, purity: 99.999%) were utilized. The sensors’ operating temperatures were regulated by connecting their meander heaters to an external power supply (Agilent U8002A, Santa Clara, CA, USA).

**Figure 2:**
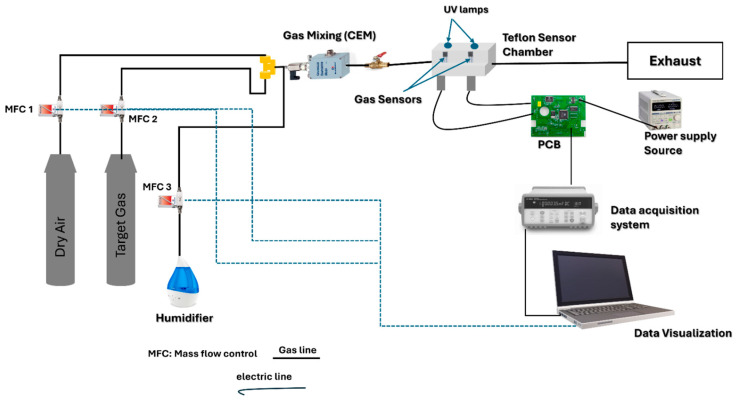
Experimental setup of gas sensing measurements.

The sensor responses were recorded using an Agilent 34972A data acquisition system, which continuously monitored the resistance of the sensing materials during exposure to varying concentrations of target gases, including NO_2_, ethanol, NH_3_, and CO_2_. Additionally, the effect of humidity on sensor performance was assessed using a controller evaporator mixer (CEM) to generate a controlled humidity level of 70% RH, simulating real environmental conditions at the optimal operating temperature. The overall gas sensing measurement setup is illustrated in Figure 2.

To optimize power consumption and simulate realistic testing conditions, the total gas flow rate was maintained at a low rate of 100 mL/min. Before measurements, the sensors were stabilized under synthetic dry air for 1 h at both room temperature and 100 °C. Following stabilization, the sensors were exposed to the target gases diluted in synthetic dry air for 15 min, followed by a 1 h recovery period in pure dry air. Throughout the measurements, the temperature inside the test chamber was maintained at 25 °C, with a residual ambient humidity of 4% relative humidity (R.H.), equivalent to approximately 1250 ppm of water vapor.

Sensor response, R (%), was defined as follows:

R (%) = [|R − R_0_|/R_0_] × 100

where R_0_ is the resistance under dry air, and R is the resistance measured during exposure to the target gas.

### 2.4. Material Characterization

The morphological characteristics of the samples were examined using scanning electron microscopy (SEM) with a FEI Quanta 450 instrument from FELMI-ZFE (Graz, Austria). Furthermore, transmission electron microscopy (TEM) imaging was performed using an ultra-high-resolution transmission electron microscope (UHR-TEM), specifically the Libra^®^ 200MC model (Zeiss, Jena, Gemrnay). Structural analysis was carried out through X-ray diffraction (XRD) measurements using the Shimadzu Corporation LabX XRD-600 instrument (Midland, ON, Canada), equipped with CuKα radiation (λ = 1.54056 Å). The XRD patterns were recorded at room temperature over a 2θ range of 10° to 80°. Raman spectroscopy was employed to analyze the structural features of the samples within a wavelength range of 100–3000 cm^−1^, using a Renishaw inVia Raman Microscope (Changchun New Industries Optoelectronics Technology Co., Ltd., Changchun, China).

## 3. Results

### 3.1. Characterization

**Figure 3:**
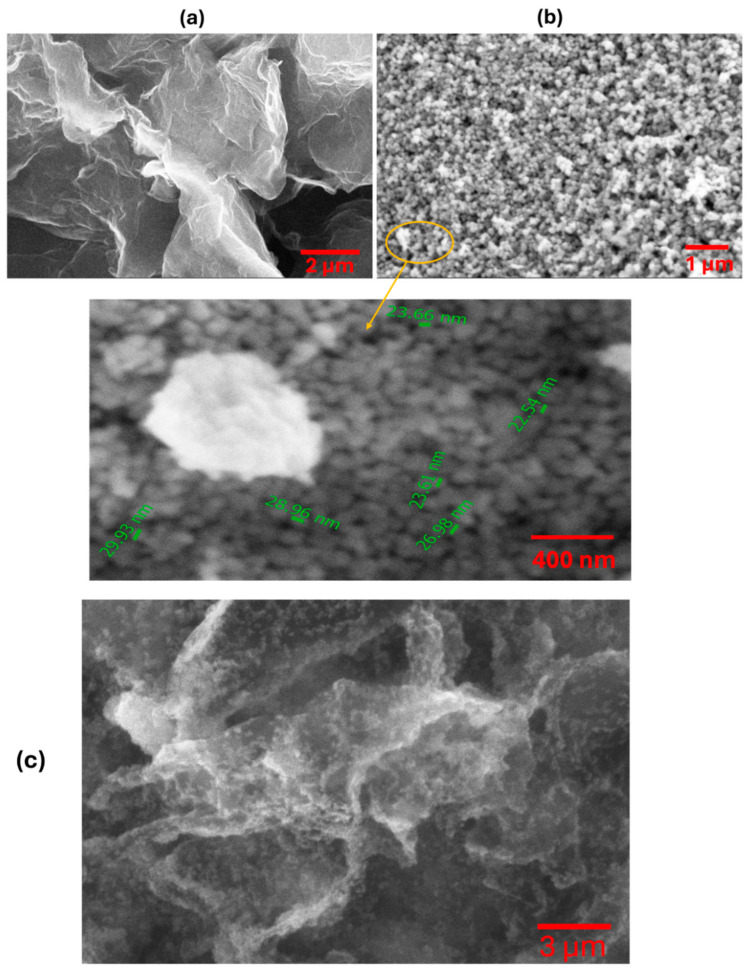
SEM images of (**a**) N-rGO and (**b**) NiO nanoparticles. (**c**) NiO/N-rGO deposited film.

SEM micrographs provide detailed insights into the morphological characteristics of N-rGO and NiO NPs and NiO/N-rGO deposited film. As shown in Figure 3a, N-rGO exhibits a distinctive folded 2D graphene flake structure, reflecting the effects of the reduction and nitrogen doping processes [14]. The observed wrinkles and corrugations are likely due to the intercalation of nitrogen atoms within the graphene layers [9]. In contrast, the NiO surface, presented in Figure 3b, reveals a distribution of spherical nanoparticles with comparable sizes. Additional details on the size distribution of NiO nanoparticles are presented in the histogram shown in Figure S2 (Supplementary Information). Figure 3c confirms the successful attachment of the deposited NiO NPs onto N-rGO sensing film. This is further supported by TEM analysis results.

**Figure 4:**
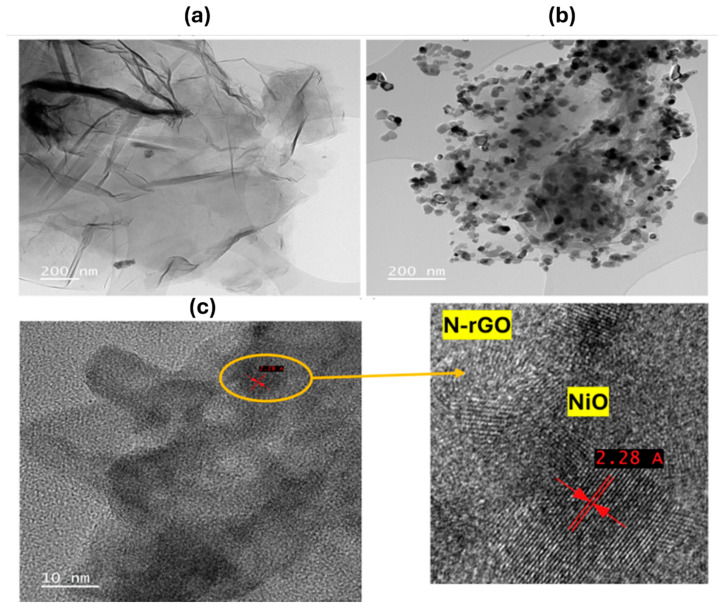
TEM images of (**a**) N-rGO and (**b**,**c**) NiO/N-rGO. The lower panel on the right shows a magnification of the circled area as indicated in panel c.

Figure 4 showcases the detailed morphological analysis of N-rGO and NiO/N-rGO using high-resolution transmission electron microscopy (HRTEM), supported by Energy Dispersive Spectroscopy (EDS) data (additional EDS results are provided in Figure S1, Supporting Information). To prepare the samples, a copper grid was immersed in a NiO/N-rGO suspension dispersed in ethanol and treated with ultrasonication to ensure even distribution.

The HRTEM image in Figure 4a reveals the characteristic two-dimensional nanostructures of graphene, displaying thin, flake-like layers folded around darker regions. In contrast, Figure 4b,c illustrates the successful and uniform decoration of nickel oxide nanoparticles across the N-rGO surface. The NiO nanoparticles exhibit a nanocrystalline structure with an interplanar spacing of 2.28 Å, corresponding to the (200) plane. This spacing, slightly offset by approximately 0.2 nm compared to the standard value (ICDD card number: 73-1519), is attributed to lattice distortions induced by interactions with the N-rGO substrate, as confirmed by XRD analysis [30].

The oxygen functional groups and nitrogen dopants present on the N-rGO surface serve as active sites for heterogeneous nucleation, enabling the formation of densely packed and monodisperse nickel oxide nanoparticles [31]. This distinctive morphological configuration of the NiO/N-rGO hybrid not only highlights the structural integrity of the material but also emphasizes its enhanced reactivity, making it a promising candidate for NO_2_ gas sensing applications.

**Figure 5:**
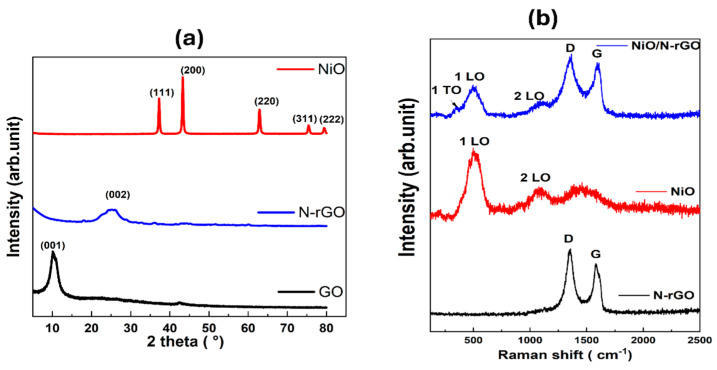
(**a**) XRD pattern of GO, N-rGO, and NiO. (**b**) Raman Spectra of N-rGO, NiO, and NiO/N-rGO.

Figure 5a shows the XRD diffractograms for the NiO, N-rGO, and GO samples. The X-ray diffraction (XRD) pattern of the nanocomposite displays distinctive broad peaks, which are localized at 37.08° (111), 43.32° (200), 62.75° (220) and 75.15° (311) [32,33,34,35]. These diffraction peaks correspond to the ones observed in the case of NiO nanoparticles. They match with a cubic phase of NiO (ICDD card number: 73-1519), with a lattice constant a = 4.168 Å belonging to the Fm-3m space [34]. Conversely, the diffractogram of GO displays a broad peak at about 2θ = 11° (001). This peak indicates the presence of oxygen functional groups in the GO structure after the oxidation of graphite [35]. Nevertheless, this distinctive peak of GO vanishes completely in the XRD pattern of the N-rGO nanomaterial, and a new characteristic peak appears at 25° (002), This means that most of the oxygen-containing groups in GO were efficiently eliminated after the reduction and doping with nitrogen [36]. This also indicates that the π-conjugated structure of graphene has been restored considerably at the produced rGO. For the NiO/N-rGO deposited film, XRD analysis was performed to examine its crystalline structure. Given the thin nature of this film on top of the alumina substrate, Al_2_O_3_ peaks highly interfere with the ones that are characteristic for NiO and N-rGO in the XRD diffractogram pattern (see Figure S3).

Raman spectroscopy, a vital technique for identifying structural fingerprints, was utilized to gain detailed insights into the structural characteristics of samples. Figure 5b depicts the Raman spectra of N-rGO, NiO, and NiO/N-rGO nanomaterials within the range of 250 to 2500 cm^−1^. The N-rGO spectrum exhibits a D band at 1339 cm^−1^ and a G band at 1570 cm^−1^ [37]. The intensity ratio of the D band to the G band (I_D_/I_G_) indicates a higher intensity of the D band, confirming the presence of structural defects associated with oxygen functional groups and nitrogen doping [9]. In the case of NiO, two prominent peaks are observed at 518 cm^−1^ and 1058 cm^−1^, corresponding to the Ni-O stretching mode (1LO) and the two-phonon vibration mode (2LO), respectively [38]. The 1LO mode represents the longitudinal optical phonon vibrations of atoms within the crystal lattice, while the 2LO mode involves the simultaneous vibration of two phonons [39]. The Raman spectrum of the NiO/N-rGO nanocomposite integrates all characteristic peaks from both NiO and N-rGO, highlighting the successful formation of the composite material. Additionally, the peak at 518 cm^−1^ is asymmetric, featuring a shoulder on the left side around 400 cm^−1^, corresponding to the 1TO peak of the first-order phonon. It is typically absent in an ideal cubic NiO structure. Its presence, however, is attributed to lattice distortions and defect states, resulting in non-stoichiometry within the Ni-O framework, such as nickel and oxygen vacancies [40].

### 3.2. Gas Sensing Characterization

**Figure 6:**
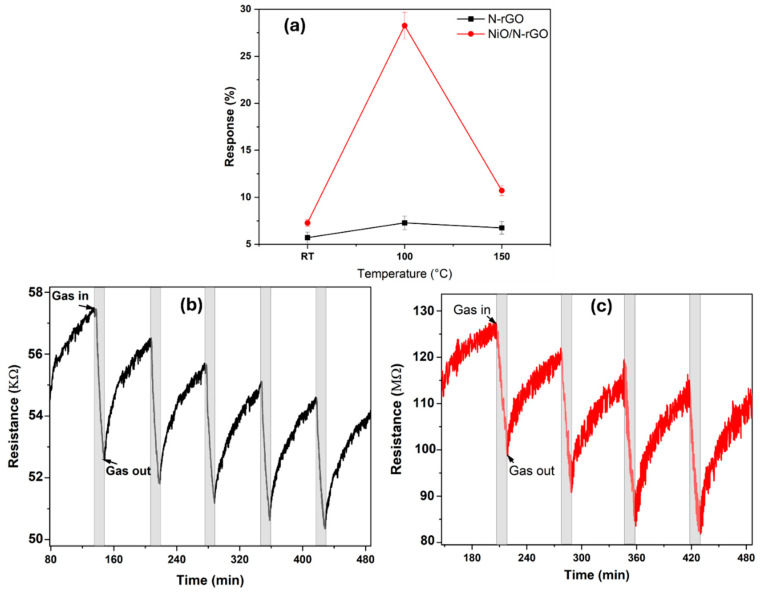
(**a**) Sensor responses as a function of their operating temperature toward 800 ppb of NO_2_. Dynamic response and recovery curves for repeated exposure cycles to 800 ppb NO_2_ for (**b**) N-rGO and (**c**) NiO/N-rGO. The sensor operating temperature was 100 °C.

The as-fabricated N-rGO and NiO/N-rGO sensors were evaluated for nitrogen dioxide (NO_2_) detection by monitoring their relative resistance changes when exposed to varying NO_2_ concentrations. As seen in Figure 6a, initial gas sensing measurements were conducted at NO_2_ concentrations of 800 ppb under room temperature (RT), 100 °C, and 150 °C conditions over multiple cycles. The results underscore the significant role of operating temperature in enhancing the sensitivity and reliability of the NiO/N-rGO sensor for NO_2_ detection. Upon exposure to NO_2_, an oxidizing gas, both sensors exhibited behavior consistent with p-type semiconductors, characterized by a decrease in resistance (Figure 6b,c). This response aligns with previous studies on NO_2_ sensing [9], where the adsorption of NO_2_ molecules onto the N-rGO surface triggers charge transfer. Specifically, NO_2_ molecules accept electrons from the N-rGO, leading to a reduction in resistance. Both sensors demonstrated stable and reproducible responses to the target gas, with effective baseline recovery after each exposure cycle.

At room temperature, while both sensors exhibited resistance changes (Figure S4), these changes were not distinguishable from baseline drift. The response magnitudes of the as-fabricated N-rGO and NiO/N-rGO sensors were calculated as 5.71% and 7.28%, respectively. This indicates that the incorporation of NiO nanoparticles into N-rGO offers limited improvement in response performance at room temperature. This modest enhancement is attributed to the high activation energy required for the NiO/N-rGO sensor [41]. In contrast, when operated at 100 °C, both sensors demonstrated significantly improved responses and a more stable resistance baseline (Figure 6b,c). The calculated responses were 7.28% for N-rGO and 28.25% for NiO/N-rGO. Notably, the NiO/N-rGO sensor exhibited a response magnitude approximately three times higher than that observed at room temperature, indicating a substantial enhancement in sensing performance. This improved response is ascribed to the low activation energy of the NiO/N-rGO sensor influenced by the rise in temperature [41]. However, at 150 °C (Figure S4), both sensors exhibited a decreased response to NO_2_ gas, further confirming that the optimal operating temperature for achieving excellent sensing performance is 100 °C. Meanwhile, the decrease observed at 150 °C may be attributed to the increased desorption rate of NO_2_ molecules from the sensor surface, which reduces the overall sensor response.

The response and recovery times were determined to be (t_resp_ = 11 min, t_rec_ = 45 min), respectively, for the NiO/N-rGO sensor and (t_resp_ = 10 min, t_rec_ = 44 min) for the N-rGO sensor, as shown in Figure S5. The response time is defined as the duration required for the sensor to reach 90% of its final stable resistance upon exposure to the target gas. Conversely, the recovery time refers to the time needed for the sensor to return to 10% of its baseline resistance after the removal of the target gas.

**Figure 7:**
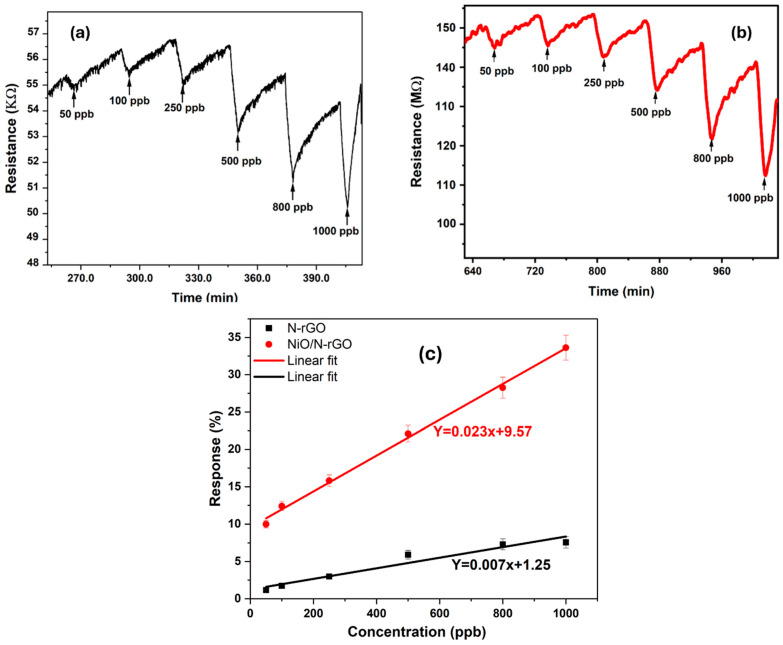
(**a**) N-rGO and (**b**) NiO/N-rGO gas sensing films’ resistance changes as a function of time toward different NO_2_ concentrations at 100 °C. (**c**) N-rGO and NiO/N-rGO sensors’ response as a function of NO_2_ concentration at 100 °C.

The dynamic resistance changes in the as-fabricated sensors were further recorded across a wide range of NO_2_ gas concentrations—50, 100, 250, 500, 800, and 1000 ppb—at an operating temperature of 100 °C, as illustrated in Figure 7a,b. The calculated responses for the N-rGO sensor were 1.18%, 1.75%, 3%, 5.9%, 7.28%, and 7.56%, respectively, while the NiO/N-rGO sensor demonstrated responses of 10%, 12.42%, 15.8%, 22.1%, 28.25%, and 33.63%, respectively. Both sensors exhibited stable and reproducible sensing responses, as evidenced by their small standard deviation errors on the order of 10^−4^ [42].

Notably, as depicted in Figure 7c, both sensors demonstrated the capability to detect NO_2_ concentrations below 50 ppb, a value significantly lower than the threshold limit of 0.2 ppm (200 ppb) for an 8 h time-weighted average (TWA) recommended by the American Conference of Industrial Hygienists (ACGIH). The NiO/N-rGO sensor showed a noticeably enhanced response for each exposure to NO_2_, outperforming the N-rGO sensor by approximately five orders of magnitude. This significant improvement highlights the remarkable influence of NiO nanoparticles on enhancing the responsiveness of the N-rGO sensing film to NO_2_ gas.

The enhanced sensing performance of the NiO/N-rGO sensor can be attributed to the large surface area of the NiO/N-rGO nanohybrids, which promotes efficient charge carrier transfer between NO_2_ molecules and the hybrid material. Additionally, XRD and Raman’s analysis confirmed the excellent crystallinity of NiO, along with the presence of oxygen vacancies, which may enhance electron mobility and strengthen NiO–NO_2_ interactions [43]. This synergistic effect highlights the significant role of NiO nanoparticles in improving gas sensing capabilities, particularly for the detection of trace levels of NO_2_.

The sensitivity and limit of detection (*LOD*) are calculated following the expression shown below:

$$LOD=3\times \frac{{RMS}_{noise}}{b}$$

where *b* is the slope of the calibration curve (sensitivity) and *RMS_noise_* is the root-mean-square deviation at the baseline [44]. *RMS_noise_* is the standard deviation of the noise level. Under dry air, the RMS noise values were determined from 100 baseline data points before NO_2_ exposure, yielding values of 9.22 × 10^−2^ for N-rGO and 0.77 × 10^−2^ for NiO/N-rGO, respectively. Consequently, the calculated limit of detection (LOD) was approximately 39 ppb for the N-rGO sensor and 0.96 ppb for the NiO/N-rGO sensor.

As demonstrated in Table 1, the as-fabricated NiO/N-rGO sensor exhibits significantly improved sensitivity and a lower limit of detection (LOD) (<1 ppb) compared to its pristine N-rGO counterpart. This enhancement underscores the pivotal role of NiO nanoparticles in augmenting the gas sensing properties of N-rGO, particularly for NO_2_ detection.

**Figure 8:**
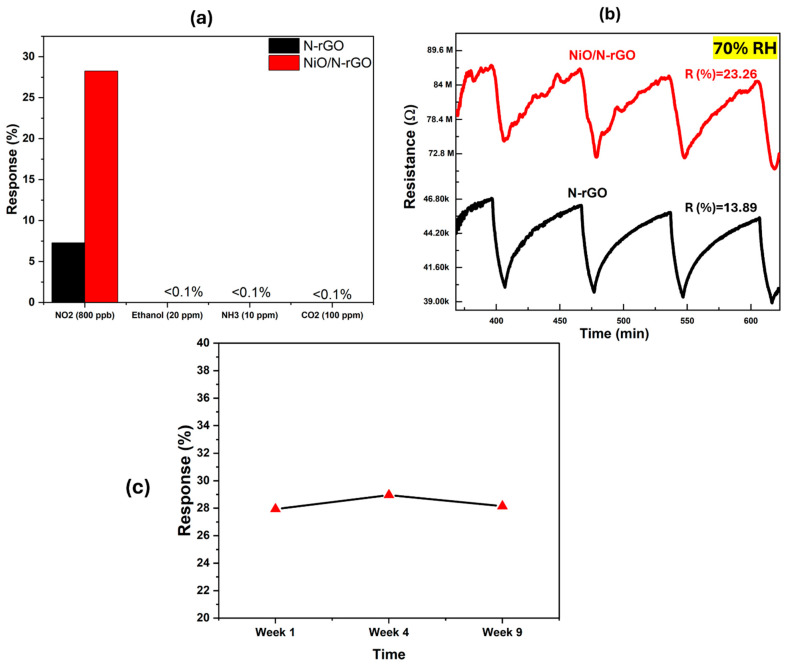
(**a**) Response histogram of N-rGO and NiO/N-rGO gas sensors to NO_2_ (800 ppb), Ethanol (20 ppm), NH_3_ (10 ppm), and CO_2_ (100 ppm). (**b**) N-rGO and NiO/N-rGO sensors’ resistance variations to 800 ppb of NO_2_ under 70% RH. Sensors operated at 100 °C. (**c**) NiO/N-rGO sensor response to 800 ppb of NO_2_ gas at 100 °C over a 9-week period.

The gas sensing performance of N-rGO and NiO-decorated N-rGO was also evaluated for other gases, including CO_2_, NH_3_, and ethanol. The typical resistance response dynamics for 100 ppm CO_2_, 20 ppm ethanol, and 10 ppm NH_3_ are presented in Figure S6, respectively (Supplementary Information). A summary of the sensing results for each gas is provided in the histogram in Figure 8a. The results clearly demonstrate that the incorporation of NiO into N-rGO significantly enhances the response to NO_2_ while effectively reducing cross-sensitivity to CO_2_, NH_3_, and ethanol. NiO was reported as a highly sensitive material for VOCs such as ethanol at high operating temperatures (<300 °C) [22]. Therefore, this temperature range is unsuitable for the current application. Operating at such high temperatures could compromise the stability of the N-rGO film and contradict the goal of achieving low power consumption for the device [45].

Ambient moisture interference is a critical parameter in evaluating the sensitivity of gas sensors under working conditions. To investigate this, the prepared sensors were tested in a highly humid environment (70% RH) at 100 °C, alongside an 800 ppb NO_2_ concentration. As shown in Figure 8b, the N-rGO sensor exhibited an increased response to ambient moisture, rising from 7.28% under dry conditions to 13.89% at 70% RH. In contrast, the NiO/N-rGO sensor showed a decreased response in the humid environment compared to its dry condition response of 23.26%.

This observation suggests that the p-type sensitivity of N-rGO improves in the presence of NO_2_, likely due to water molecules promoting the adsorption of NO_2_ through redox reactions. During this process, nitrogen dioxide dissociates into NO_2_^−^ and H^+^ ions, facilitating the movement of H^+^ protons. This proton mobility decreases the resistance, resulting in an enhanced electrical response [46]. On the other hand, the notable decrease in sensitivity for the NiO/N-rGO sensor under high humidity can be attributed to two factors. Firstly, the presence of water molecules inhibits the chemisorption of oxygen molecules by covering the surface of NiO/N-rGO with adsorbed moisture. Secondly, the interaction of water molecules with oxygen species leads to the formation of hydroxyl groups on the sensor surface, which partially hinders the adsorption of NO_2_ molecules. These factors significantly alter the sensor’s resistance and diminish its response to NO_2_ gas in a highly humid environment [47,48].

All measurements were conducted over a period of 9 weeks in which, even though a drift appeared in the value of the baseline resistance (see Figure S7 in Supporting Information), no significant changes were observed in sensor responses (see Figure 8). This confirms the stability of both N-rGO and NiO/N-rGO sensors in NO_2_ detection.

As presented in Table 2, the as-fabricated NiO/N-rGO sensor exhibits a markedly superior response to trace levels of NO_2_ gas compared to previously reported MO/graphene-based sensors. This improvement emphasizes the effectiveness of the synergistic interaction between nitrogen-doped reduced graphene oxide and nickel oxide nanoparticles in enhancing NO_2_ gas sensing performance.

## 4. Discussion

**Figure 9:**
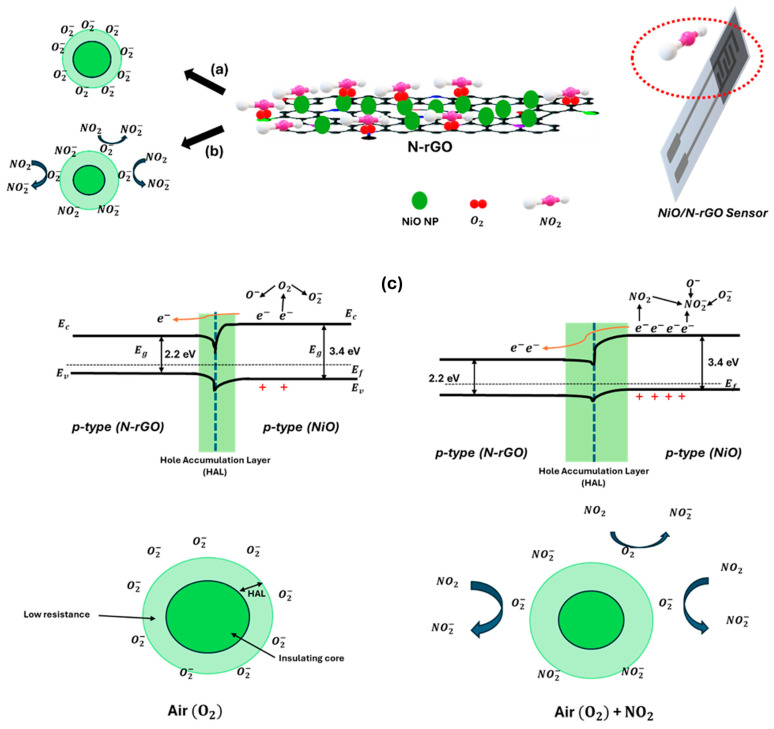
Formation of core–shell structures of charge carriers in NiO to (**a**) air (O_2_), (**b**) NO_2_, and (**c**) the sensing mechanism of NiO/N-rGO sensitivity to NO_2_ at 100 °C. At the interface between p-type N-rGO and p-type NiO, a Hole Accumulation Layer (HAL) forms in both cases under air or in the presence of NO_2_.

The interaction mechanism between the NiO/N-rGO sensing film and NO_2_ gas molecules is further elucidated in Figure 9.

To begin, it is essential to describe the systematic interaction between pristine N-rGO and NO_2_ molecules. Upon exposure to an oxidizing gas like NO_2_, the resistance curves exhibit a p-type response, consistent with the intrinsic behavior of reduced graphene oxide (rGO) as a p-type semiconductor [55]. Nitrogen doping, as an element from Group V, enhances n-type conduction in rGO by incorporating pyrrolic, pyridinic, and graphitic bonds within the graphene basal plane and donates one p-electron to the aromatic π system [10]. This nitrogen doping introduces excess electrons, promoting stronger interactions with NO_2_. Furthermore, nitrogen atoms can bind with the oxygen atoms of NO_2_ molecules [56]. In contrast, this study demonstrates that N-rGO exhibits a p-type response toward NO_2_, confirming its p-type semiconducting behavior. This phenomenon can be attributed to the interaction between NO_2_ molecules and the aromatic π-system of N-rGO, where nitrogen dopants (as verified by EDS analysis) play a crucial role. It is hypothesized that NO_2_ molecules withdraw electrons from the π-system at the nitrogen-doped sites, thereby reinforcing the p-type semiconducting behavior of N-rGO. As a result, when N-rGO reacts with NO_2_, its Fermi level shifts closer to the valence band, increasing the number of holes in the valence band and thus causing a significant decrease in sensor resistance upon exposure to nitrogen dioxide.

Turning to the NiO/N-rGO sensing film mechanism, Figure 9 illustrates the sequential interactions. Initially, as shown in Figure 9a, when the sensing film is exposed to air, oxygen molecules are adsorbed onto the surface of NiO nanoparticles (NPs). At an operating temperature of 100 °C, these oxygen molecules are ionized into O_2_^−^ and O^−^ species. On the p-type NiO surface, these ionized oxygen species attract the majority carriers (holes), forming a well-defined Hole Accumulation Layer (HAL).

In the presence of NO_2_ gas, as depicted in Figure 9b, the adsorbed NO_2_ molecules act as strong electron acceptors and capture electrons from the p-type NiO, forming NO_2_^−^. This process significantly increases the hole concentration in the NiO NPs, leading to an expansion of the HAL. Consequently, the potential barrier height decreases due to the high electron affinity of NO_2_, which surpasses that of oxygen.

Furthermore, as illustrated in Figure 9c, the hybrid configuration of N-rGO sheets and NiO nanoparticles facilitates the formation of a p-p junction. N-rGO may donate electrons to the NiO NPs, enhancing the binding with NO_2_ and O_2_ and further widening the depletion layer [57]. Additionally, nitrogen-doped rGO contains active sites, such as nitrogen dopants not bound to NiO NPs, which provide further interaction opportunities with NO_2_ gas. N-rGO also serves as an efficient charge transport channel due to its high carrier mobility, enabling faster electron transfer to the electrodes for collection.

These attributes collectively endow the NiO/N-rGO nanohybrids with exceptional NO_2_ sensing performance and enhanced sensitivity, stability, and carrier transport efficiency.

## 5. Conclusions

In summary, we have successfully prepared a NiO/N-rGO nanohybrid-based NO_2_ gas sensor based on an alumina transducer substrate using facile and inexpensive routes. The sensing materials were widely investigated for their main structures and analyzed using SEM, TEM, XRD, and Raman techniques. Interestingly, NiO NPs were well attached to the surface of N-rGO, giving insights into the sensing mechanism, as confirmed by characterization results. Accordingly, the NiO/N-rGO sensor exhibited excellent sensitivity toward trace amounts of nitrogen dioxide (NO_2_) at a moderate operating temperature of 100 °C. This represents the first demonstration of a high-yield synthesis technique that produces NiO/N-rGO nanohybrids, which can be readily deposited on a wide range of substrate materials. In the first test, the sensor showed a very high selectivity toward NO_2_ (the other gaseous species tested were CO_2_, ethanol, and NH_3_). Consequently, NiO NPs proved their potential for boosting the sensitivity of N-rGO toward NO_2_ gas, thanks to the p-p junctions created that facilitate carrier conduction, as explained by the underlying sensing mechanism. The nanomaterial presented robust performances such as a high sensitivity and very low limit of detection, showing high prospects for being integrated in the next generation of advanced chemoresistive sensors.

## Figures and Tables

**Table 1:** Sensitivity and LOD values.

Sensors	N-rGO	NiO/N-rGO
Sensitivity (10^−2^ ppm^−1^)	709	2398
LoD (ppb)	39	<1

**Table 2:** Comparison of the performance in the detection of NO_2_ between this work and previously reported results.

Material-Based Sensor	T (°C)	NO_2_ (ppm)	Response (%)	Tresp/Trec (s)	LOD (ppb)	Ref.
NiO NPs/N-rGO	100	0.8	28.25	660/2700	<1	This work
N-rGO	100	0.8	7.28	600/2640	39	This work
SnO_2_/rGO	150	100	97.24	14/509	1000	[49]
NiO NS/rGO	200	1	~670	-	-	[50]
ZnO/rGO	110	2.5	33.11	182/234	5	[51]
NiO Honeycomb	200	20	57.3	-	20	[52]
CuO/rGO	RT	20	58.1	30/-	1000	[53]
ZnO/SnO_2_/rGO	RT	5	141	33/92	-	[54]

## Data Availability

Data can be obtained from the authors upon request.

## Supplementary Materials

The following supporting information can be downloaded at: https://www.mdpi.com/article/10.3390/s25051631/s1. Figure S1: EDS analysis of (a,b) N-rGO and (c,d) NiO/N-rGO; Figure S2: size distribution histogram of NiO nanoparticles; Figure S3: XRD pattern of N-rGO, NiO, and NiO/N-rGO; Figure S4: Resistance curves of N-rGO and NiO/N-rGO sensors toward 800 ppb of NO_2_ at room temperature and 150 °C; Figure S5: Response and recovery times of N-rGO and NiO/N-rGO sensors toward 800 ppb of NO_2_ at 100 °C; Figure S6: Resistance curves of N-rGO and NiO/N-rGO sensors toward 20 ppm ethanol, 100 ppm of CO_2_, and 10 ppm NH_3_ at 100 °C; Figure S7: Dynamic response and recovery curves of the NiO/N-rGO sensor over 9 weeks of measurements. Repeated exposures to 800 ppb NO_2_ at an operating temperature of 100 °C.
