# Supplementary material for: Synergistic Enhancement of Chemiresistive NO2 Gas Sensors Using Nitrogen-Doped Reduced Graphene Oxide (N-rGO) Decorated with Nickel Oxide (NiO) Nanoparticles: Achieving sub-ppb Detection Limit
Source: Sensors (Basel). 2025 Mar 6;25(5):1631. doi: 10.3390/s25051631 (PMC11902665; doi:10.3390/s25051631)
Supplement: Supplementary file 1 [file sensors-25-01631-s001.zip › sensors-3494475-supplementary.pdf]

Supporting information for:

Synergistic Enhancement of Chemiresistive NO_2 Gas Sensors Using Nitrogen-Doped Reduced Graphene Oxide (N-rGO) Decorated with Nickel Oxide (NiO) Nanoparticles: Achieving sub-ppb Detection Limit

Chiheb Walleni [1,2,3,4,5], Mounir Ben Ali [5,6], Mohamed Faouzi Ncib [4,5] and Eduard Llobet [1,2,3,*]

[1] MINOS, School of Engineering, Universitat Rovira i Virgili, Avda. Països Catalans 26, 43007 Tarragona, Spain; chiheb.walleni@estudiants.urv.cat

[2] IU-RESCAT, Research Institute in Sustainability, Climatic Change and Energy Transition, Universitat Rovira i Virgili, Joanot Martorell 15, 43480 Vila-seca, Spain

[3] TecnATox—Centre for Environmental, Food and Toxicological Technology, Universitat Rovira i Virgili, Avda. Països Catalans 26, 43007 Tarragona, Spain

[4] Higher School of Sciences and Technologies of Hammam Sousse, University of Sousse, Hammam Sousse 4011, Tunisia; mohamed.faouzi.ncib@gmail.com

[5] NANOMISENE Laboratory, LR16CRMN01, Center of Research on Microelectronics and Nanotechnology (CRMN), Technopole of Sousse, B.P334, Sahloul 4054, Tunisia; mounirbenali@crmn.mesrs.tn

[6] Higher Institute of Applied Science and Technology of Sousse, University of Sousse, Sousse 4003, Tunisia

* Correspondence: eduard.llobet@urv.cat; Tel.: +34-977-558-502

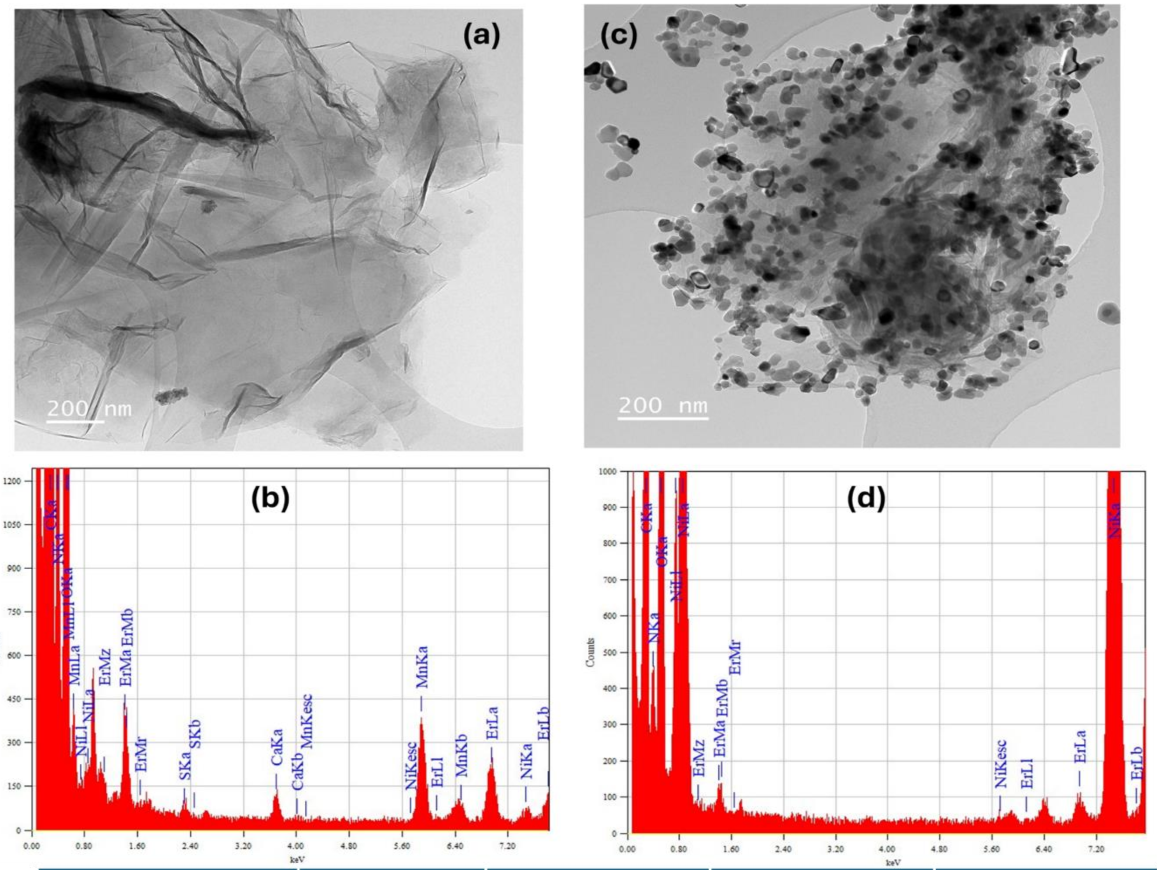

Samples	Carbon (%)	Oxygen (%)	Nitrogen (%)	Nickel (%)
**N-rGO**	91.6	5.34	3.06	-
**NiO/N-rGO**	53.02	26.13	2.19	18.65

Figure S1. EDS analysis of (a,b) N-rGO and (c,d) NiO/N-rGO.

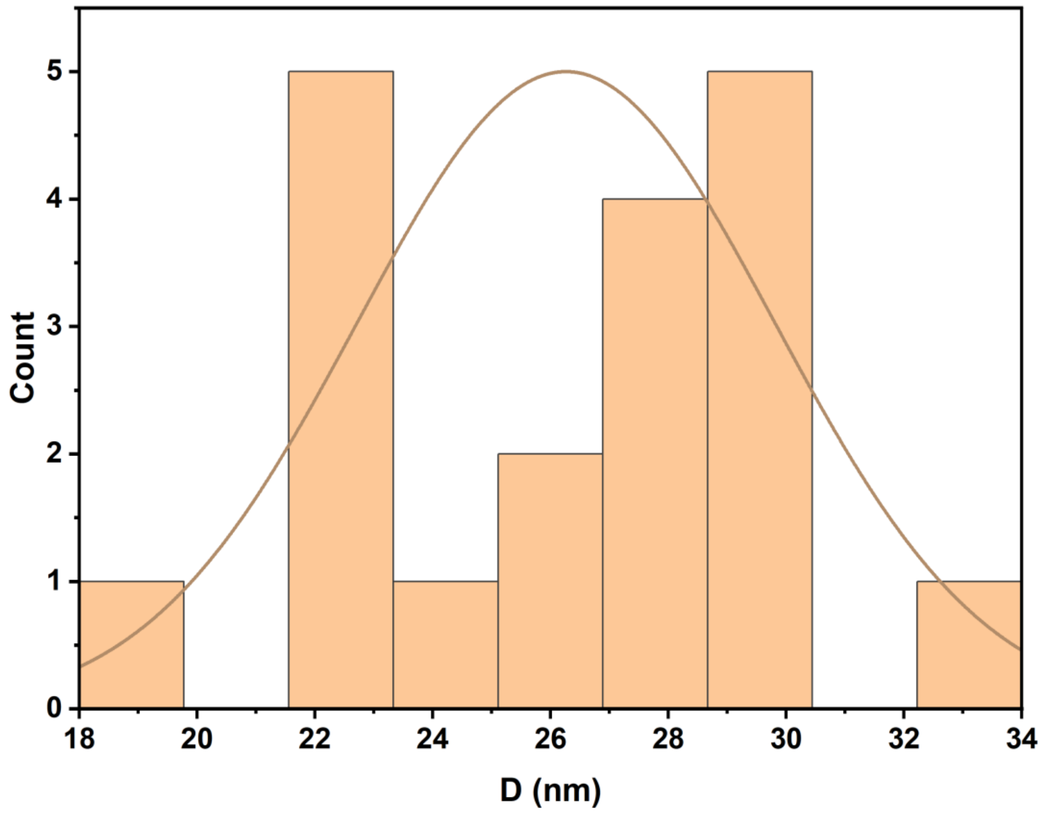

Figure S2. Size Distribution Histogram of NiO Nanoparticles

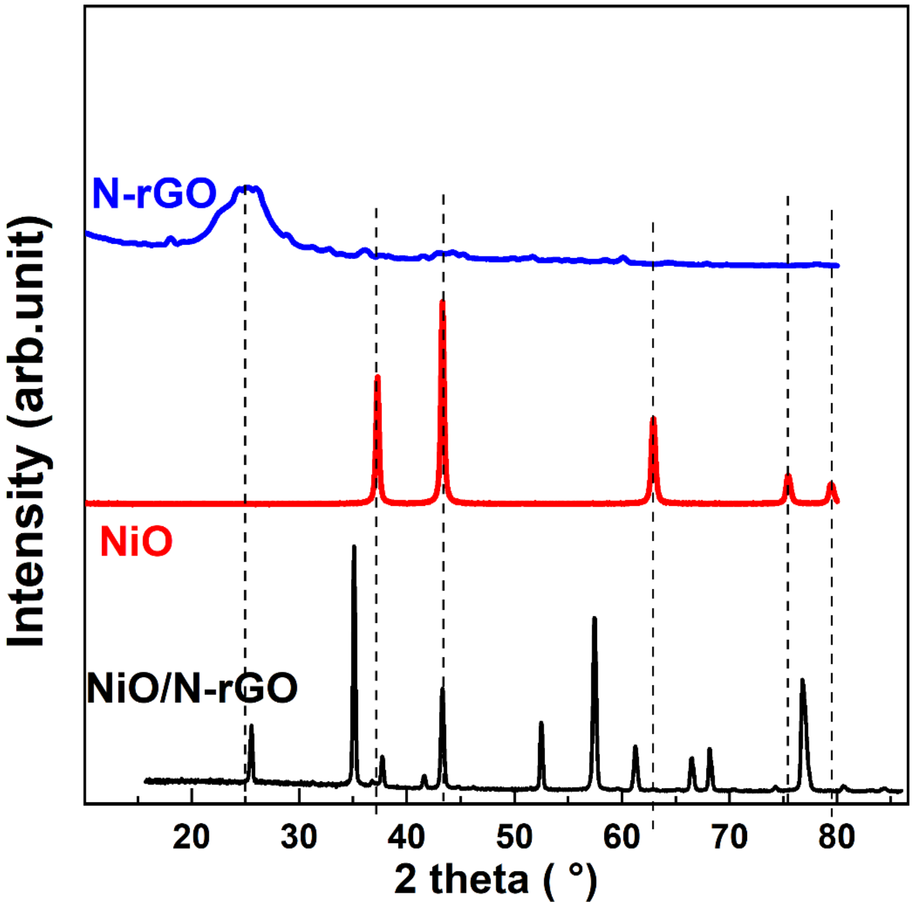

Figure S3. XRD pattern of N-rGO, NiO, and NiO/N-rGO.

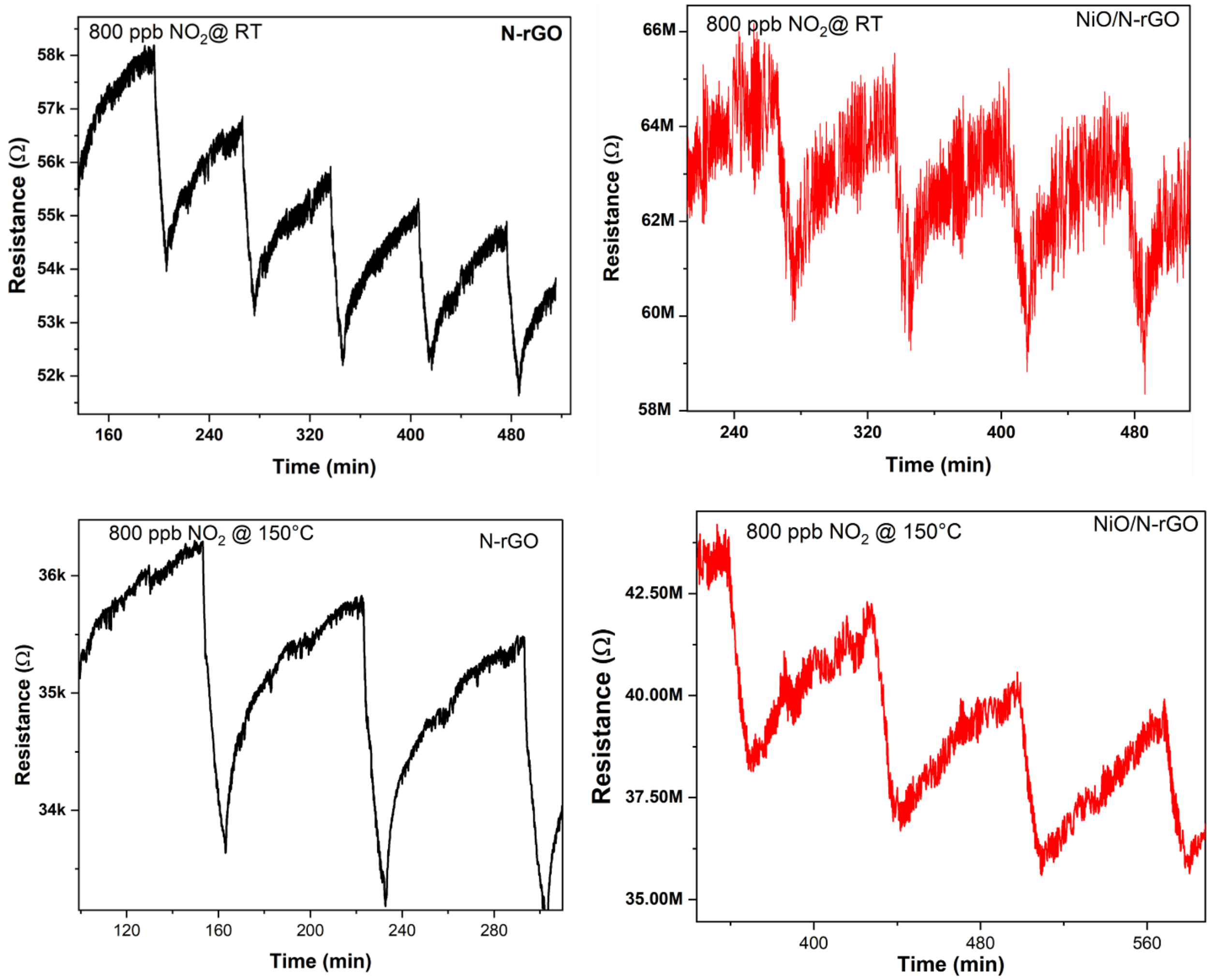

Figure S4. Resistance curves of N-rGO and NiO/N-rGO sensors toward 800 ppb of NO_2 at room temperature and 150°C.

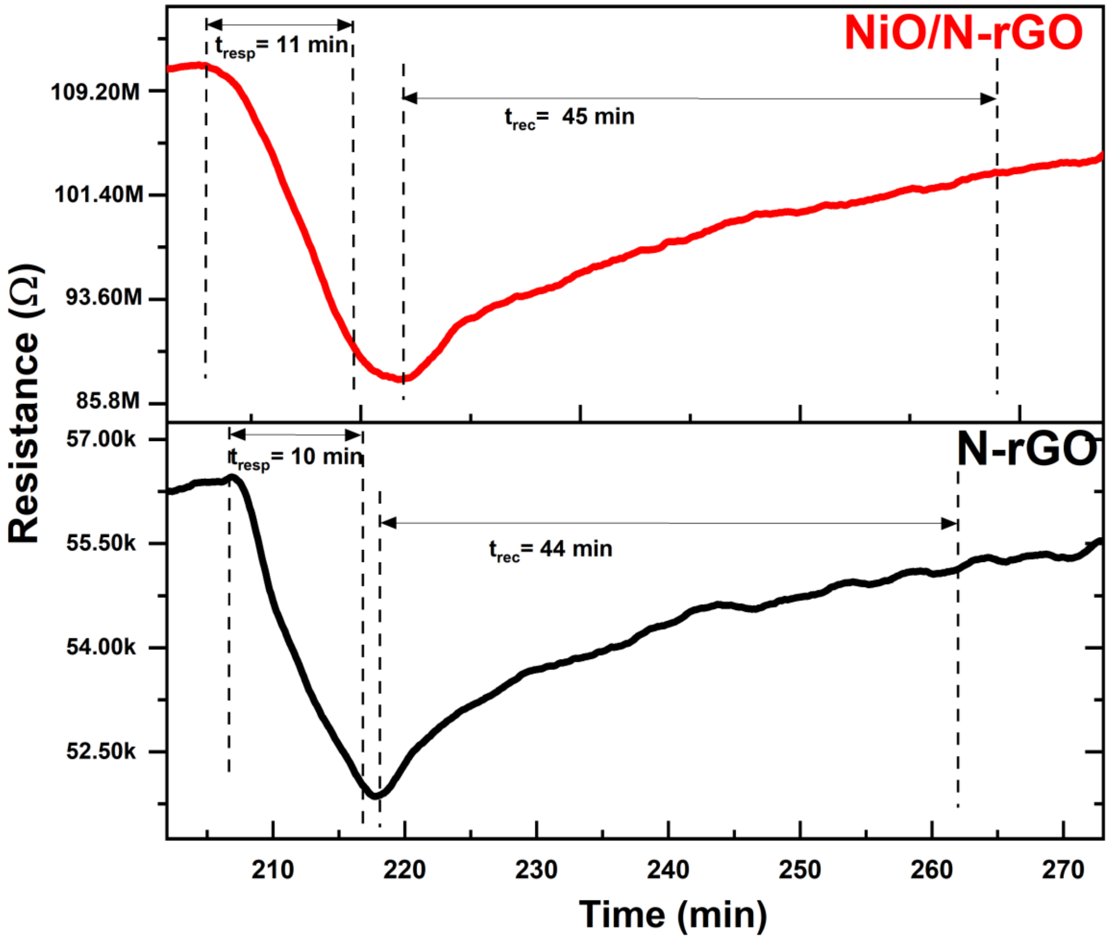

Figure S5. Response and recovery times of N-rGO and NiO/N-rGO sensors toward 800 ppb of NO_2 at 100 °C.

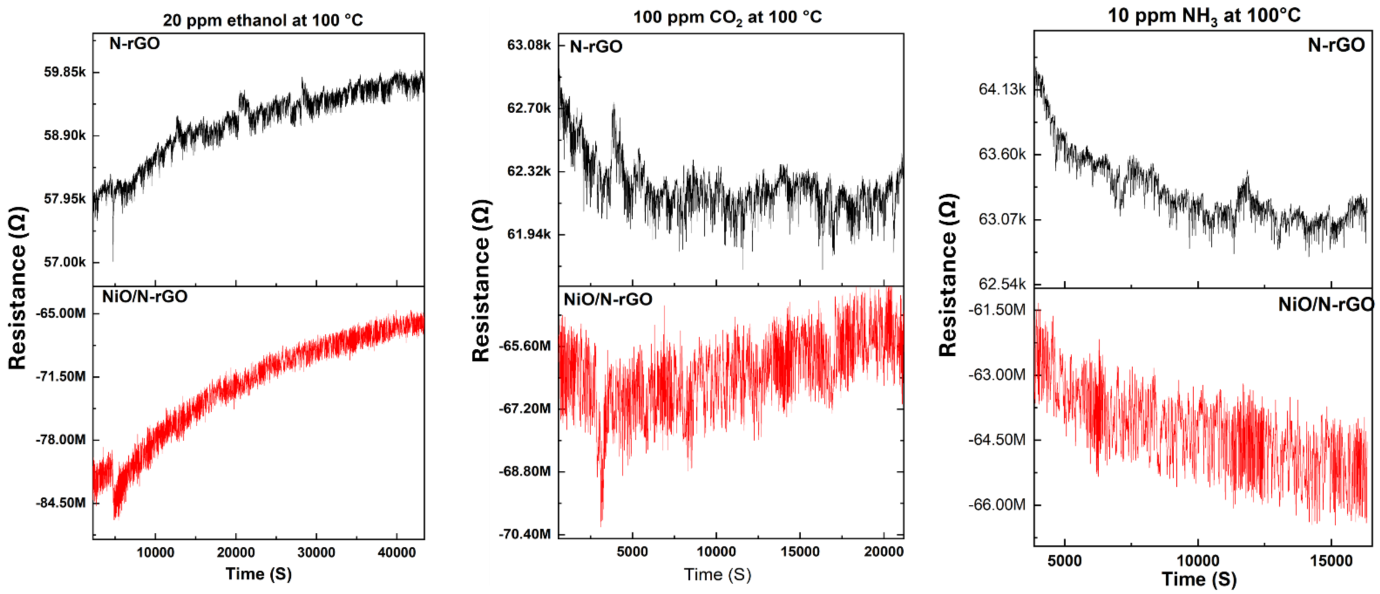

Figure S6. Resistance curves of N-rGO and NiO/N-rGO sensors toward 20 ppm ethanol, 100 ppm of CO_2, and 10 ppm NH_3 at 100 °C

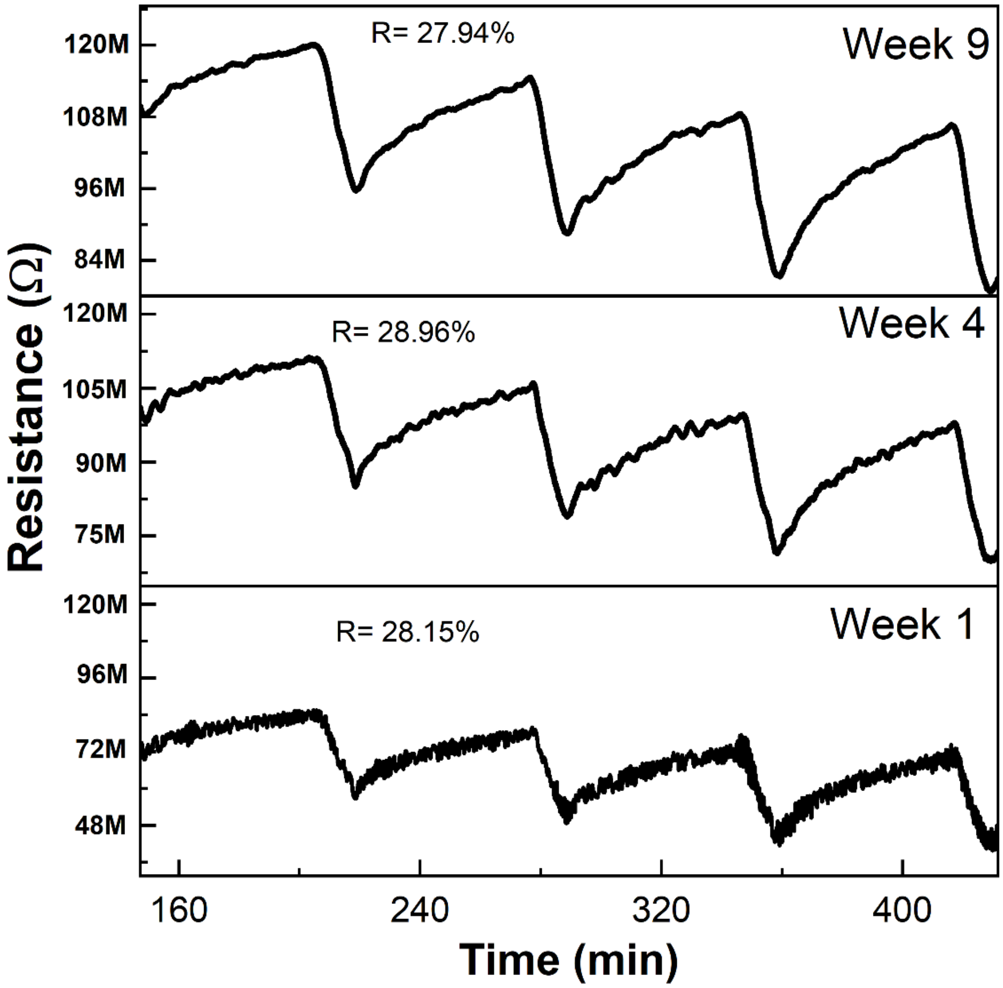

Figure S7. Dynamic response and recovery curves of the NiO/N-rGO sensor over 9 weeks of measurements. Repeated exposures to 800 ppb NO_2 at an operating temperature of 100°C.
